# Mapping the behaviour change potential of meal kits to positively influence parental food literacy

**DOI:** 10.1017/S136898002300263X

**Published:** 2023-12-01

**Authors:** Kylie Fraser, Brittany J Johnson, Penelope Love, Alison Spence, Rachel Laws, Karen J Campbell

**Affiliations:** 1 School of Exercise & Nutrition Sciences, Deakin University, Melbourne Burwood Campus, 221 Burwood Highway, Burwood, VIC 3125, Australia; 2 Caring Futures Institute, Flinders University, Bedford Park, SA, Australia; 3 College of Nursing and Health Sciences, Flinders University, Bedford Park, SA, Australia; 4 Institute for Physical Activity and Nutrition (IPAN), Deakin University, Melbourne Burwood Campus, 221 Burwood Highway, Burwood, VIC 3125, Australia

**Keywords:** Behaviour change techniques, Behaviour change wheel, Theoretical domains framework, Mechanisms of action, Food literacy, Parents, Meal kits

## Abstract

**Objective:**

This study aimed to examine the theoretical potential of meal kit subscription services in Australia to promote parental food literacy using the retrospective application of behaviour change frameworks.

**Design:**

A one-week subscription was purchased for all Australian-based meal kit subscription services (*n* 9) to access content and features available to subscribers. Behaviour change techniques (BCTs) identified in the subscription and meal planning features, meal kit delivery (i.e. ingredients and recipes) and website were coded using the behaviour change technique taxonomy (BCTTv1) and associated behaviour change frameworks. Identified BCTs were mapped to the theoretical domains framework to identify potential mechanisms of action for influencing parental food literacy development.

**Setting:**

Australia.

**Results:**

Thirty-five BCTs were identified across the nine meal kit services reviewed, ranging from nineteen to twenty-nine BCTs per company. Sixteen BCTs were common to all meal kits services, from the hierarchical clusters of ‘goals and planning’, ‘shaping knowledge’, ‘social support’, ‘natural consequences’, ‘comparison of behaviour’, ‘repetitions and substitution’, ‘associations’, ‘reward and threat’, ‘antecedents’ and ‘regulation’. Across the meal kit services, the most frequently identified mechanisms of action were motivation (*n* 27) and capability (*n* 19).

**Conclusion:**

These findings support the applicability of behaviour change frameworks to commercial meal kit subscription services and provide a theory-informed process for identifying BCTs that may be relevant for promoting parental food literacy within this context. Further research is required to explore how families engage with meal kit subscription services to determine the exposure and delivery of identified BCT content and to evaluate the potential influence on food literacy development.

Cooking and eating at home provide opportunities to improve public health nutrition and promote healthy dietary habits^(1)^. The consumption of frequent home-cooked meals has been associated cross-sectionally with a higher diet quality in both adults^(2,3)^ and children^(4,5)^. However, trends in many countries, including Australia, suggest that families are relying heavily on foods prepared outside the home including pre-prepared convenience meals and fast food^(6–8)^ in response to perceived time scarcity, fatigue and a lack of skills to plan, prepare and eat family meals^(9,10)^. Ultra-processed convenience meals^(11)^, intended to be eaten at home but requiring little to no additional preparation before consuming, are increasingly available in Australian supermarkets^(12)^. Such food procurement strategies are likely to reduce the healthfulness of the family diet^(13)^, as foods prepared outside the home are generally of lower nutritional quality and associated with increased daily energy intake^(14)^ and weight gain^(15)^.

Improving food literacy may support families to cook healthy meals, given evidence that a high level of food literacy is associated with a higher diet quality and capacity to overcome perceived barriers^(16)^. The concept of food literacy as defined by Vidgen and Gallegos^(17)^ is *‘a set of inter-related knowledge, skills and behaviours required to plan, manage, select, prepare and eat food to meet needs and determine intake’* (p.54). Food literacy established in childhood provides protective benefits for diet quality and builds the ‘scaffolding’ required to facilitate healthy dietary behaviours across the life course^(17)^. Parents play an important role in fostering children’s food literacy, with the home environment a major influence on the development of children’s food-related knowledge, skills and behaviours.

Exploration of interventions that could enhance parental food literacy and equip parents with strategies, which may improve family diet quality is warranted. Interventions aimed at improving cooking and food skills (i.e. meal planning, food acquisition and budgeting) have reported positive outcomes for diet quality, cooking confidence and knowledge^(18)^. These interventions have tended to focus on individual components of food literacy, targeting nutrition knowledge, cooking and food skills development (e.g. meal planning and budgeting)^(18)^, highlighting a missed opportunity to incorporate food literacy more holistically. Additionally, many food and cooking skills interventions are resource intensive and require out of home attendance limiting their scalability.

A novel approach to improving food literacy and supporting parents to overcome obstacles to family meal provisioning may be through meal kit subscription services (MKSSs). The global MKSS industry has increased substantially in the past decade, including the emergence of a growing number of companies in Australia^(19)^ and internationally^(20)^. These services enable cooking at home through the delivery of ‘meal kits’, conveniently boxed, containing pre-measured/semi-prepared ingredients paired with recipes. In addition, they provide an online platform for consumers to pre-order meals from a wide selection of cuisines and meal types (e.g. vegetarian, ‘kid/family friendly’) on a weekly basis. MKSSs reportedly appeal to ‘time-poor’ consumers who desire convenience and ease in selecting and preparing home-cooked meals by reducing the time and effort associated with meal planning, shopping and cooking^(21)^. Meal kits users have been previously characterised as working professionals and families^(19,22)^ who may have high household incomes and educational attainment^(23)^. However, the emergence of lower price point MKSSs may improve the affordability and accessibility of these services to populations with greater budgetary restraints.

There is speculation that MKSSs may promote healthy dietary behaviours (i.e. cooking at home from scratch, shared meals) and improve vegetable intakes^(22,24–27)^ by reducing barriers and increasing capacity for healthy meal provisioning. Two studies conducted in New Zealand reported that meal kits, sourced from commercial MKSS, My Food Bag^TM^ and Bargain Box^TM^, provided to participants at no-cost had a positive impact on dietary behaviours (i.e. more frequent home cooking, shared meal preparation) and emotional well-being of families with adolescents^(24,25)^. A recent qualitative study with Australian families (*n* 16) with children 0–18 years of age reported that MKSSs were perceived as valuable in reducing perceived barriers such as decision-making fatigue, mental load and lack of help^(23)^. Moreover, MKSSs were reported to improve cooking skills and confidence of all family members resulting in wider diet variety and intakes^(23)^. Furthermore, research has recently begun to explore the potential of providing participants with non-commercial, tailor-made meal kits (i.e. ingredient and recipe boxes) to reduce barriers to food access and improve aspects of food literacy. For example, non-commercial meal kits have been incorporated in interventions to support low-income families to implement more frequent home-cooked family meals^(28)^, to improve accessibility and facilitate at home food preparation of healthy foods for families experiencing food insecurity^(29,30)^ and to improve food literacy and diet quality of college students who cook infrequently^(31)^. Despite growing interest in meal kits as an intervention to improve health and dietary outcomes, a theory-based understanding of *how* and *why* meal kits may elicit behaviour change, and opportunities to increase their behaviour change capability, is currently lacking.

This study aimed to examine the theoretical potential of MKSSs in Australia, as a hypothetical intervention to promote parental food literacy behaviours using the retrospective application of behaviour change and food literacy frameworks. These findings will provide insight into the opportunities that exist for meal kits to be harnessed as a tool to promote healthy family food provisioning, enhance food literacy and promote the development of healthy dietary behaviours. Furthermore, identifying and specifying the behavioural content of MKSSs may provide potential learnings for community-based meal kit interventions to support families with limited income experiencing food insecurity.

## Methods

### Study design

This study used a cross-sectional design to identify MKSSs in Australia and the retrospective application of behaviour change frameworks to MKSS content and features. This research was underpinned by a pragmatic research paradigm^(32)^. Pragmatism emphasises an action-focused perspective in exploring real-world problems in terms of their practical functioning by using the most appropriate research methods for answering the research question^(32)^. Prior to conducting this study, it should be acknowledged that KF, a health promotion researcher and parent, had used MKSSs intermittently over the previous 4 years and had recently conducted a qualitative study exploring families’ experiences of MKSSs^(23)^.

### Underpinning theoretical frameworks

Behaviour change frameworks provide theory-based approaches to understand and characterise interventions. There are numerous frameworks available. For this study, we selected the behaviour change wheel (BCW) and associated behaviour change technique taxonomy (BCTTv1) and theoretical domains framework (TDF). The BCW is a comprehensive and coherent model based on a synthesis of nineteen frameworks of behaviour change^(33)^, underpinned by the capability, opportunity, motivation and behaviour (COM-B) model^(33)^. The COM-B model can be further elaborated into fourteen theoretical domains using the TDF. The TDF integrates 128 theoretical constructs from thirty-three health behaviour change theories to provide a more detailed understanding of the determinants of a given behaviour^(33)^. The BCW represents the latest advances in the field of behaviour change science as shown by its frequent use in the design^(34–36)^ and evaluation^(37)^ of a diverse range of health-related behaviours. The BCW provides a mechanism to identify intervention options (i.e. ‘intervention functions’) to target the required behaviour change using specific behaviour change techniques (BCTs). BCTs are the smallest features (‘active components’) of an intervention that have the potential to bring about a change in behaviour^(33)^. To provide a standardise approach to the application of BCT, Michie and colleagues developed the BCT taxonomy consisting of ninety-three distinct BCTs grouped into sixteen categories^(38)^. Together these frameworks (BCW and BCTTv1) can be applied retrospectively to intervention content to understand *‘how’* (BCT) an intervention works to influence behaviour and *‘why’* (theoretical mechanisms of action, TDF) this occurs.

To quantify and map the potential of MKSSs to influence parental food literacy development, Vidgen and Gallegos’s^(17)^ food literacy framework (including four domains and eleven components) was used to derive specific parental behaviours that may be addressed by MKSSs. Vidgen and Gallegos’s conceptualisation of food literacy is currently the most widely cited and comprehensive definition of food literacy^(39)^.

### Meal kit subscription services sample and inclusion criteria

A structured search using Google Chrome and Boolean logic was conducted in March 2022 to identify all commercially available MKSSs operating in Australia. Search terms included a combination of the following: ‘meal kits’, ‘food boxes’, ‘recipe box’, ‘delivery’ and ‘subscription services’. Meal kits were defined as a subscription service that provides predominately fresh, pre-measured ingredients, paired with recipes, delivered directly to consumers to prepare at home. Services that did not involve home meal preparation and cooking (i.e. pre-prepared or ready-to-heat meals) were excluded. MKSSs operating in Australia were identified (*n* 9), and a 1-week subscription was purchased for each provider by KF (between May-June 2022). Three meals for two people were ordered from each MKSS to gain access to content and features only available through a paid subscription. As the nutritional content of recipes and meal plans (i.e. number of meals and servings per person) were not relevant to the research question, meals were chosen based on the author’s personal preference. Due to the geographical restrictions of the areas serviced by MKSSs (i.e. not all were available in each Australian State/Territory), KF was the recipient of five meal kit boxes, and two researchers (AS and a research assistant) received a meal kit from the remaining four MKSSs (*n* 1 and *n* 3 respectively). Subscriptions to each MKSS enabled KF to document users’ ‘meal kit experience’ and subscription functions for each MKSS, including subscriber website and recipe content, emails, notifications and the delivery of a meal kit.

### Data/information sources

Data were collected from several sources during the 1-week subscription period. These included MKSS websites (i.e. general content, meal planning and selection features, blogs, FAQ and embedded videos), mobile phone apps, personalised emails and notifications and meal kit delivery (i.e. meal kit packaging, ingredients, recipes and promotional content). Screenshots of emails, text messages and notifications were downloaded and saved for analysis. The three researchers (KF, AS and research assistant) who received meal kits utilised a standarised protocol to document key features and content (e.g. packaging, recipe cards and additional promotional content). Data reported from meal kit deliveries were extracted into a purpose-designed Microsoft Excel spreadsheet. Excluded from the analysis were any MKSS social media accounts, video advertisements, emails and promotion materials (including discount vouchers) that were available after cancelling the one-week subscription.

### Procedure

**Fig. 1:**
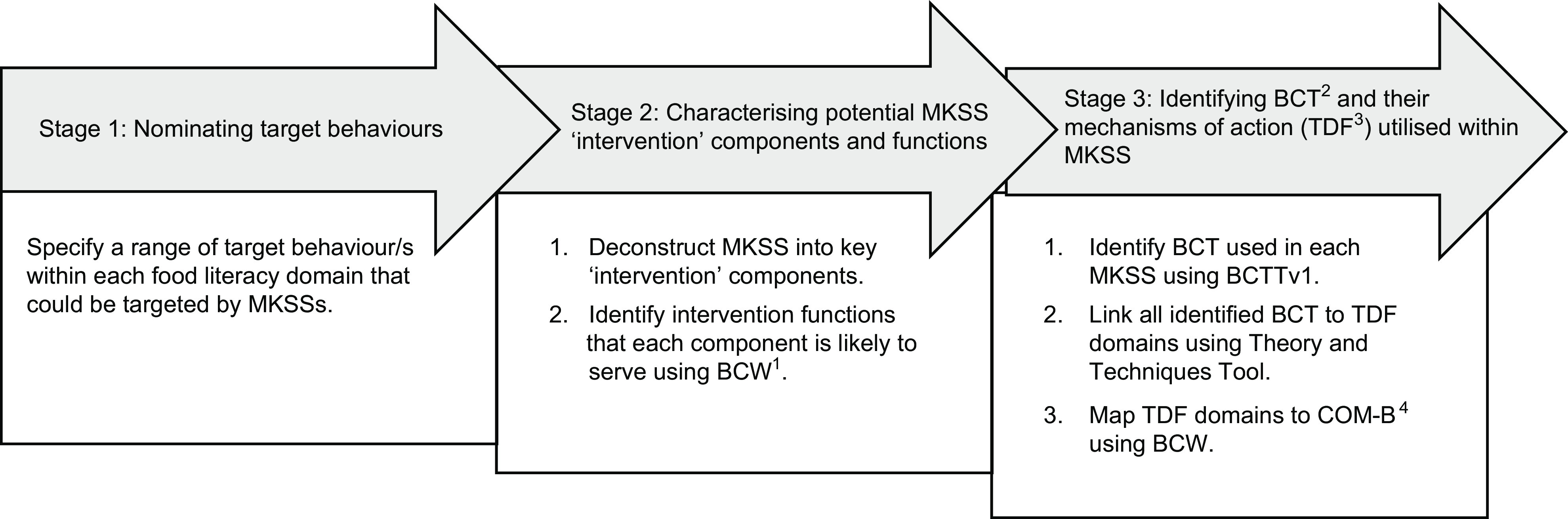
^1^Behaviour change wheel (BCW). ^2^Behaviour change techniques (BCTs). ^3^Theoretical domains framework (TDF). ^4^Capability, opportunity and motivation model of behaviour (COM-B)

Figure 1 outlines the three-stage process and steps involved in the retrospective application of the food literacy^(17)^ and behaviour change frameworks^(33,38)^ to MKSS content.

### Stage 1: Nominating target behaviours

Vidgen and Gallegos’s^(17,40)^ food literacy framework was used to derive specific parental target behaviours that may be influenced by MKSSs. This involved specifying the four food literacy domains and eleven components^(17,40)^ in behavioural terms to understand ‘what’ food literacy looks like in terms of specific behaviours. Consensus on final parental food literacy behavioural clusters was agreed by three authors (KF, BJJ and PL).

### Stage 2: Characterising potential meal kit subscription services ‘intervention’ components and functions

MKSS content identified during data collection was characterised into three key ‘intervention’ components (i.e. subscription and meal planning features, delivery of a meal kit and website content) common to all MKSSs. These components were mapped to the BCW intervention functions by KF using the BCW Guide to Designing Interventions^(33)^ and experience using MKSSs. Primary functions were identified as the main purpose of the MKSS component, whereas secondary functions were considered as supporting or additional functions. Two members of the research team (BJJ, PL) with experience applying behaviour change frameworks and knowledge of the MKSS context reviewed the mapping. Consensus on the likely functions that each MKSS component served was developed in agreement between all three researchers (KF, BJJ and PL).

### Stage 3: Identifying behaviour change techniques and their mechanisms of action (theoretical domains framework) utilised within meal kit subscription services

#### Behaviour change technique coding and analysis

A pragmatic approach informed the coding of BCTs in MKSS sources guided by standard coding procedures outlined by Michie et al^(38)^. Best practice reporting guidelines for retrospective BCT taxonomy application were followed (i.e. detail coders experience, independence and coding process, report identified BCT numbers and labels)^(41)^. Two members (KF, BJJ) of the research team, with formal training in BCT taxonomy application (https://www.bct-taxonomy.com/), coded MKSS sources between July–August 2022. KF had recent experience coding content from an early life nutrition and movement behaviour intervention using the BCTTv1. BJJ is an experienced coder and has coded intervention content from a range of projects including interventions to reduce parent unhealthy food provision, mobile apps to support healthy family food provision and early childhood obesity prevention trials.

Due to the novel nature of meal kits and application of the BCTTv1 to this context, a codebook was developed by KF to provide coding guidelines specific to this context. This involved reviewing MKSS content alongside the ninety-three BCTs and definitions, with the aim of identifying examples from MKSS content to create additional coding rules to provide greater clarity to the coding process. Areas of ambiguity were resolved via discussion with BJJ on two separate occasions, prior to finalising the codebook. To ensure coding consistency, one MKSS company (i.e. Marley Spoon^TM^) was randomly selected for independent coding in duplicate (KF, BJJ). BCTs were reported in a Microsoft Excel spreadsheet using ‘Yes’, ‘No’ or ‘Maybe’ to indicate the presence, absence or likelihood of a BCT. Coders also recorded where the BCT was identified (i.e. which MKSS source) and a direct ‘excerpt’ as evidence. Only BCTs that were relevant to the target behaviour clusters were coded.

Following coding, inter-coder agreement was assessed using prevalence-adjusted and bias-adjusted (PABAK) score^(42)^. PABAK was used as it controls for high levels of negative agreement often present in BCT coding. A PABAK value of 0·87 was observed for identifying BCTs in the dual-coded MKSS, which is classified as ‘excellent/almost perfect’ agreement^(43)^. KF independently coded the remaining MKSSs (*n* 8) and discussed coding ambiguities (i.e. ‘maybe’ codes) with BJJ and KC. The coding instructions were further refined to provide examples specific to the meal kit context. For example, BCT 1·2 Problem solving was redefined to include examples of text from MKSSs that included an identifiable barrier and solution that resonates with the target population (i.e. common barriers families face to planning, shopping and preparing homecooked meals). BJJ completed a cross-check of all BCT codes to finalise the coding process. No changes were made to the final BCT coding post cross-checking.

#### Mapping Behaviour change techniques to mechanisms of action (theoretical domains framework)

BCTs were mapped to the fourteen TDF domains and corresponding COM-B elements (i.e. physical and psychological capability, physical and social opportunity and reflective and automatic motivation) using the Theory and Techniques Tool to identify the theoretical mechanisms of action^(44)^. The Theory and Techniques Tool is an interactive matrix that triangulates data from a synthesis of published literature and expert consensus to depict the strength of the association (link) between seventy-four BCTs and twenty-six mechanisms of action (including the fourteen TDF domains) (https://theoryandtechniquetool.humanbehaviourchange.org/)^(44)^. The mapping exercise was undertaken by KF to identify the theoretical links and strength of association between BCTs and TDF domains identified in MKSS components using the interactive Theory and Techniques Tool resource. The mapping exercise (i.e. matrix) was reviewed by BJJ and PL. Uncertainties in links were discussed until consensus was reached by applying both a theoretical perspective (i.e. Theory and Techniques Tool mapping) and a practical perspective (i.e. drawing on our knowledge of MKSS context and the potential mechanisms of action we judged BCT to serve). In some cases, BCTs with TDF domain links were identified by the research team despite insufficient evidence of an established link in the tool.

### Data synthesis

Key characteristics of MKSSs and intervention functions for each MKSS component were narratively synthesised and presented descriptively. BCTs identified across the MKSSs (i.e. unique BCTs) are reported, and BCTs common to all MKSSs are synthesised and reported by MKSS component. Links between BCTs and TDF domains are reported for MKSS components and include all instances of identified BCTs (i.e. some BCTs were present in more than one MKSS component).

## Results

Nine MKSSs were identified as operating across Australia, including four multinational companies and five Australian-based companies. Four MKSSs operated Australia-wide and five were restricted to specific metropolitan areas and/or Australian States/Territories. The MKSSs included common features such as meal planning, ordering and delivery. However, they differed in the amount and type of content they provided on their recipes (e.g. pictures of preparation/cooking steps and links to social connectivity/support), website information and blogs (e.g. persuasive marketing and promotions and/or health and nutrition-related information).

### Nominated target behaviours

Target behaviours were specified and grouped into clusters of related parental behaviours under the four food literacy domains as defined by Vidgen and Gallegos^(17,40)^, namely plan and manage, select, prepare and eat. Examples of specific parental behaviours clustered under the four food literacy domains are detailed in Table 1.

**Table 1:** Examples of specific parental behaviours for each food literacy domain

Food Literacy Domains*	Examples of specific parental behaviours
Plan and manage *Ability to plan and make decisions regarding food intake that balance needs (nutrition, taste and hunger) and available resources (time, money, skills and equipment) so that food can be regularly accessed.*	Plan and manage time and budget for food purchasing. Plan meals and eating occasions in advance. Plan for regular meal routines (including providing home-cooked meal and eating together). Plan for meals to minimise food waste and/or limit impulsive food purchases by utilising shopping lists. Plan meals to include appropriate types of foods (e.g. vegetables, meat and meat alternatives, dairy, fruit and wholegrains) and seasonal foods. Involve children and other family members in the planning, procurement and preparation of meals. Plan meals to meet a range of needs (e.g. nutrition, taste and hunger) and available resources (e.g. skills and equipment).
Select *Knowledge and understanding of food including where it comes from, quality, how to use and store, to meet needs.*	Gather or access food from different sources (e.g. supermarkets, farmers markets and online). Purchase and provide a wide variety of appropriate types of foods (e.g. vegetables, meat and meat alternatives, dairy, fruit, wholegrains). Select and purchase mostly fresh ingredients for preparation at home and understand where they came from and how to use them. Limit use of ultra-processed foods in provisioning of meals (e.g. frozen/microwave foods and meals, read-to-heat sauces, takeaways).
Prepare *Knowledge and skills to prepare a meal using basic food safety hygiene, commonly available cooking equipment and foods. Ability to follow or adapt recipes to meet food needs with available resources (skills, time and equipment).*	Prepare a wide variety of meals using common pieces of kitchen equipment, utensils and appliances. Prepare a wide variety of meals using commonly available foods (e.g. vegetables, meat and meat alternatives, dairy, fruit and wholegrains). Prepare home-cooked meals using mostly fresh or minimally processed ingredients. Prepare and store food appropriately and safely. Follow and/or adapt written recipes to meet needs (e.g. nutrition, taste) with available resources (including kitchen equipment, skills and time).
Eat *Knowledge and understanding of nutrition concepts (e.g. foods for good health, portion sizes) including the impact food and eating together has on health and well-being.*	Provide regular meal routines (including eating together, eating same meal). Provide regular home-cooked meals. Provide appropriate portion sizes of each food group, within a meal and/or across eating occasions over a period of time. Offer a wide variety of nutritious and balanced meals regularly.

* Food Literacy Domains and definitions replicated from Vidgen & Gallegos^(17,40)^.

### Intervention functions incorporated in meal kit subscription services

Eight of the nine BCW intervention functions (i.e. education, persuasion, incentivisation, coercion, training, environmental restructuring, modelling and enablement) were identified across the MKSS components. Each MKSS component was identified as serving both primary and secondary functions (see online supplementary material, Supplemental Material 1). The BCW function not featured was *restriction* (i.e. rules to restrict engagement in the target behaviour or unwanted behaviours by changing the external environment).

Within the subscription and meal planning component, *enablement* was identified as the primary (main) function of the MKSS, supported by secondary (additional) functions *coercion* (i.e. paid subscription/contract), *incentivisation* (i.e. free or discounted subscription for user and family/friend) and *training* (i.e. imparting meal planning skills). The meal kit delivery component provided the primary functions of *environmental restructuring* and *enablement*, with supporting functions identified as *education* (i.e. step-by-step recipes, environmental consequences of reducing food waste), *training* (i.e. facilitates the preparation and cooking of meals at home using recipe) and *modelling* (i.e. inclusion of pictures on recipes modelling preparation/cooking method). The website content, including general MKSS information, pictures and images, customer reviews, FAQ and blogs, provided the primary functions of *education* and *persuasion* (i.e. to increase knowledge and change beliefs/encourage action towards using MKSSs), with *environmental restructuring* identified as a secondary function based on messaging and images that encouraged children/partners involvement in preparing and cooking meal kits.

### Presence of behaviour change techniques in meal kit subscription services

Across the nine MKSS reviewed, thirteen of the sixteen BCT categories were represented and thirty-five of the ninety-three BCTs within these categories identified (Table 2). The number of BCTs present in each MKSS ranged from nineteen to twenty-nine, with sixteen BCTs common to all MKSSs (see online supplementary material, Supplemental Material 2 for details of BCT identified for individual MKSS). A total of twenty-four BCTs were present in more than half (*n* ≥ 5) the MKSSs. Eleven unique BCTs were present in less than half (*n* ≤ 4) the MKSSs. Of the sixteen BCTs present across all MKSSs, seven BCTs were incorporated through the subscription and meal planning component, six were identified in the meal kit delivery component and three through textual information or images on MKSS websites. Table 3 summarises the sixteen common BCTs used in each MKSS component and intervention functions used.

**Table 2:** Frequency of identified behaviour change techniques (BCTs) across nine Australian meal kit subscription services (MKSSs)

	Number of MKSS incorporating BCT	
BCT categories, number and labels (*n* 35 identified)	*n*	%
1. Goals and planning		
1·1 Goal-setting (behaviour)	9	100
1·2 Problem solving	5	55
1·4 Action planning	9	100
1·8 Behavioural contract	9	100
1·9 Commitment	9	100
2. Feedback and monitoring		
No BCT identified for this category	0	
3. Social support		
3·1 Social support (unspecified)	8	88
3·2 Social support (practical)	9	100
4. Shaping knowledge		
4·1 Instruction on how perform behaviour	9	100
4·2 Information about antecedents	2	22
5. Natural consequences		
5·1 Information about health consequences	7	77
5·2 Salience of consequences	9	100
5·3 Information about social and environmental consequences	9	100
5·6 Information about emotional consequences	3	33
6. Comparison of behaviour		
6·1 Demonstration of the behaviour	7	77
6·2 Social comparison	1	11
6·3 Information about others’ approval	9	100
7. Associations		
7·1 Prompts and cues	9	100
8. Repetition and substitution		
8·1 Behavioural practice/rehearsal	9	100
8·2 Behaviour substitution	6	66
8·3 Habit formation	9	100
9. Comparison of outcomes		
9·1 Credible source	7	77
9·2 Pros and cons	3	33
10. Reward and threat		
10·1 Material incentive (behaviour)	7	77
10·2 Material reward behaviour	4	44
10·3 Non-specific reward	9	100
10·4 Social reward	1	11
10·6 Non-specific incentive	2	22
11. Regulation		
11·2 Reduce negative emotions	5	55
11·3 Conserving mental resources	9	100
12. Antecedents		
12·1 Restructuring the physical environment	9	100
12·2 Restructuring the social environment	1	11
12·3 Avoidance/reducing exposure to cues for the behaviour	1	11
12·5 Adding objects to the environment	9	100
13. Identity		
13·2 Framing/reframing	2	22
14. Scheduled consequences		
No BCT identified for this category	0	
15. Self-belief		
15·1 Verbal persuasion about capability	1	11
16. Covert learning		
No BCT identified for this category	0	

**Table 3.** Behaviour change techniques (BCTs) and intervention functions of the most common BCTs (*n* 16) identified in meal kit subscription service (MKSS) components

MKSS component	Description of MKSS component	BCT*	Evidence of BCT in MKSS components	Intervention functions†
Subscription and meal planning	Written and financial agreement of subscription, personalised emails/text notifications, subscription perks for friend referrals (i.e. send a free box), meal planning feature that allows subscriber to select meals at least one week in advance for a set number of people and days per week and automatic pre-selection of meals based on personal preferences.	1·1 Goal-setting (behaviour)	Allows users to set goals in terms of meal planning behaviours by requiring them to subscribe to a meal plan that includes a set number of people and days per week. Also promotes meal planning through ordering at least one week in advance.	**Enablement** Coercion Incentivisation Training
		1·4 Action planning	Prompts detailed planning of cooking at home behaviours by requiring users to select meals in advance for a set number of people and days per week.	
		1·8 Behavioural contract	Subscription service enters users into a written agreement (financial commitment).	
		1·9 Commitment	Subscription service that enters users into a written agreement (financial commitment made).	
		7·1 Prompts and cues	Subscribers are sent an email notifying of upcoming delivery and pantry requirements, or to select meals for next week with the purpose of cueing and promoting behaviour.	
		10·3 Non-specific reward	Subscription perks for referrals (i.e. send a free box to family/friends).	
		11·3 Conserving mental resources	MKSS prepopulating recipes based on preferences and automatic selections based on previous orders to reduce mental resources and facilitate behaviour change.	
Meal kit delivery	Box of ingredients and recipes (hard copy or online access), as well as any promotional/marketing material delivered directly to the subscribers’ doorstep.	3·2 Social support (practical)	The provisioning of meal kits (i.e. ingredients and recipes) to users doorstep is a form of practical support for the performance of cooking at home.	**Enablement** **Environmental restructuring** Education Training Modelling
		4·1 Instruction on how to perform the behaviour	MKSS websites provide detailed instructions on how to plan, select and order meals. Recipes provide detailed food preparation and cooking instructions.	
		8·1 Behavioural practice/rehearsal	Prompt practice of food preparation one or more times in the context of cooking a meal at home.	
		8·3 Habit formation	Automatic subscription – prompts to encourage maintenance of behaviour.	
		12·1 Restructuring the physical environment	Provision of meal kit box with ingredients and recipes changes the physical environment in order to facilitate performance of food preparation and consumption behaviours.	
		12·5 Adding objects to the environment	Delivery meal kit box with ingredients and recipes facilitate performance of food preparation and consumption behaviours.	
Website content	All information accessible on the website such as general MKSS information (i.e. ‘About Us’, ‘What we do’), FAQ (i.e. answers to general questions) and blogs.	5·2 Salience of consequences	Pictures/advertising designed to emphasise and bring attention to the performance of food preparation, cooking and consumption behaviours to make the consequences more memorable.	**Education** **Persuasion** Environmental restructuring
		5·3 Information about social and environmental consequences	Written, verbal and visual images of environmental consequences (e.g. reduce food waste) and social consequences (e.g. locally sourced and sustainable food supply, % of ingredients sourced from Australia) of performing the behaviour of planning and purchasing meals from MKSS.	
		6·3 Information about others’ approval	Customer reviews on meal kit website provide information of what other people think (their approval) of using meal kits to perform target behaviours (meal plan and manage, select, prepare and eat).	

Primary intervention functions are **bold**.

* BCT definitions as defined in the behaviour change technique taxonomy (BCTTv1)^(38)^.

† Intervention functions as defined in the behaviour change wheel (BCW)^(33)^.

### Linking behaviour change techniques to mechanisms of action (theoretical domains framework)

The ‘intervention’ components and functions of MKSSs (*n* 9) linked to all six elements of the COM-B (i.e. physical and psychological capability, physical and social opportunity and reflective and automatic motivation), particularly reflective motivation (*n* 27) and psychological capability (*n* 19). Overall, the BCTs identified across each MKSS component linked to thirteen of the fourteen TDF domains (see online supplementary material, Supplemental Material 3). The most frequently identified links between BCTs and TDF domains across the MKSS components were knowledge (*n* 9, psychological capability), beliefs about consequences (*n* 9, reflective motivation), behavioural regulation (*n* 8, psychological capability), social influences (*n* 8, social opportunity), environmental context and resources (*n* 8, physical opportunity) and beliefs about capabilities (*n* 8, reflective motivation).

Within the subscription and meal planning component, the most frequently identified links between BCTs and TDF were reinforcement (*n* 4, automatic motivation) and intentions (*n* 3, reflective motivation). The BCTs identified in the meal kit delivery component were most frequently linked to knowledge (*n* 4, psychological capability), environmental context and resources (*n* 4, physical opportunity) and beliefs about capabilities (*n* 3, reflective motivation). The BCTs identified in the website content were most frequently linked to beliefs about consequences (*n* 7, reflective motivation), knowledge (*n* 4, psychological capability), behaviour regulation (*n* 4, psychological capability) and social influences (*n* 4, social opportunity).

## Discussion

This study aimed to examine the theoretical potential of commercially available MKSSs in Australia, as a hypothetical intervention, to promote parental food literacy using the retrospective application of behaviour change and food literacy frameworks. Overall, the behaviour change content of MKSSs linked to all six COM-B elements and included thirty-five of a potential ninety-three BCTs (nineteen to twenty-nine BCTs per MKSS), eight of nine intervention functions and thirteen of the fourteen TDF domains. This suggests that MKSSs may have the potential to influence parental food literacy through several pathways.

Overall, our findings highlight that MKSSs incorporate two main intervention functions – enablement and environmental restructuring. These intervention functions may support parents to overcome opportunity- and motivation-related barriers to cooking at home such as time poverty, low energy and perceived need for help/support^(9,10)^. Through these functions, MKSSs may support the streamlining of family meal provisioning and reshape the home environment to enable cooking and eating at home with reduced effort and mental resources. Qualitative research with MKSS users in Australia^(23)^ and Denmark^(45)^ has previously reported that meal kits reduced perceived time constraints and mental effort required to plan and prepare family meals. Education, training and modelling functions were identified to a lesser degree but may influence parental capability by imparting knowledge and skills to support family meal provisioning.

It is both interesting and promising that commercial MKSSs incorporated a suite of BCT categories that have the potential to influence parental food literacy. Existing evidence suggests that some BCTs identified in MKSSs may be effective for influencing aspects of food literacy^(46)^ and dietary intake^(47)^. Of the thirty-five unique BCTs identified, five (*BCT 4·1 instruction on how to perform behaviour, 5·1 and 5·3 information about health, social and environmental consequences, 6·1 demonstration of behaviour and 8·1 behavioural practice/rehearsal)* are frequently used in adult food and cooking skills interventions, particularly interventions reporting long-term (> 3 months) behaviour change^(46)^. Furthermore, thirteen of the thirty-five BCTs were reported as potential effective agents of change in a meta-analysis of dietary interventions targeting young adults (17–35 years)^(47)^. In particular, *BCT 5·2 salience of consequences, 8·3 habit formation and 12·5 adding objects to the environment* were reported in this review as important BCTs for influencing dietary behaviour change^(47)^.

Our findings highlight that across MKSSs only sixteen BCTs were commonly used, with a minimum of nineteen BCTs in any one MKSS, indicating an opportunity for the inclusion of less frequently used or untapped BCTs in this context. Most of the BCTs identified, (i.e. eighteen of thirty-five BCTs) were incorporated in the website component of MKSSs (i.e. general content, FAQ and blogs); however, only three BCTs were commonly identified. Since it is unknown whether people engage in the website content, it may be more valuable for MKSSs to include these BCTs in personalised emails or at time of purchase to increase the likelihood of influencing food literacy-related behaviours. Although more BCTs is not necessarily better^(47)^, there is room for optimising the potential of MKSSs by incorporating BCTs specifically targeting aspects of food literacy.

Three BCT clusters (i.e. feedback and monitoring, scheduled consequences and covert learning) were not identified in the MKSS context. Although scheduled consequences and covert learning are not likely to be useful in this context, there is potential to explore the BCT cluster representing feedback and monitoring. Emerging evidence suggests that the inclusion of self-regulatory BCTs, such as those from ‘feedback and monitoring’ and ‘goals and planning’ clusters, is associated with greater effectiveness in nutrition/dietary behaviour change interventions^(48,49)^. In the context of MKSSs *BCT 2·2 feedback on behaviour* could be used to target automatic motivation through the mechanism of action ‘reinforcement’. For example, personalised emails could notify subscribers of the number of home-cooked meals they have prepared, the cooking skills they have used or feedback on reaching dietary guidelines such as percentage of daily vegetables consumed and/or the number of different vegetables included across their week.

Identifying the BCTs in MKSS components enables us to understand ‘how’ these core features may influence food literacy. Linking these BCTs to their underlying mechanism of action (TDF domain) enhances our theoretical understanding of ‘why’ these changes may occur. Our findings suggest that the components of MKSSs may influence behaviour collectively through mechanisms of action targeting change primarily in reflective motivation, psychological capability and opportunity (social and physical). The most frequent mechanisms of action through which the BCTs in MKSSs may elicit behaviour change include knowledge, behavioural regulation, physical and social opportunity and beliefs about consequences and capabilities. Understanding the links between BCTs identified in MKSSs and mechanisms of action is important for intervention development and evaluation of meal kit interventions. Furthermore, these findings can be used to identify underutilised or unexplored BCTs that could be incorporated in a meal kit intervention to target specific mechanisms of action.

For example, in addition to providing instructions (*BCT 4·1*) and demonstration of steps using pictures (*BCT 6·1*), MKSS recipes could include BCTs to influence the social environment (social opportunity) of family mealtime practices to target the ‘preparation and eat’ food literacy domains. This could be achieved by incorporating text/messaging to promote role modelling (*BCT 13·1 identification of self as a role model*) and including children in food preparation practices (*BCT 3·1 social support practical, 12·2 restructuring the social environment*). Current evidence strongly supports that mealtime practices such as parental role modelling of healthy eating, involving children in meal preparation and consuming the same family meal positively influence children’s eating behaviours^(50)^.

Although not specifically designed to influence dietary practices, our findings suggest that MKSSs may have the potential to positively influence parental food literacy development. Enhanced food literacy may support families to overcome perceived barriers to healthy family food provisioning and strengthen diet quality^(17)^. Moreover, MKSSs may have a positive spillover effect on the food literacy-related behaviours of other family members. Recent qualitative research with Australian families reported that meal kits facilitated opportunities for children’s active participation in meal preparation and cooking which was perceived to enhance their food-related skills, knowledge and confidence^(23)^. Children’s early involvement in food-related tasks may in turn facilitate the consumption of a wider dietary variety and intakes through increased familiarity, exposure and hands-on experiences with food^(51)^. Further research is required to explore how families engage with MKSSs to determine the exposure and delivery of BCT content identified in MKSSs and potential influence on food literacy development. Moreover, it would be useful to explore commercial perspectives to understand the opportunities for building BCTs into MKSSs to positively influence consumer food literacy and dietary behaviours.

### Implications and further research

This is the first study to provide a theoretical understanding of how and why the provision of ‘meal kits’ (i.e. ingredient and recipe bundles) may influence food literacy behaviour change. This has important implications for future research, particularly given the increasing interest in meal kits as an intervention strategy^(24,25,28–31)^. To our knowledge, no meal kit interventions have incorporated BCTs and many lack theoretical underpinnings. Designers of interventions could use these findings as a resource to identify and select BCTs to influence specific mechanisms of action to enhance the behaviour change potential of meal kits as an intervention strategy.

Research suggests MKSS cost may be a barrier to use, limiting their reach to low-income families^(23,26,52)^. Furthermore, access to and use of digital technology presumes a general level of literacy and resources, which may be an additional barrier to commercial MKSSs in promoting food literacy more broadly. Notwithstanding, our findings provide insight into the potential of commercial meal kits to influence food literacy behaviours and yield learnings that may be applied to community and food relief organisations. For example, our findings may be of interest to non-commercial sectors including community hubs to support disadvantaged populations by providing free or low-cost meal kits. Several studies have reported that tailor-made healthy meal kits are feasible and acceptable among food insecure and low-income families to overcome barriers to family meal provisioning^(29,30,52)^. Our findings may assist programme developers to understand the behaviour change potential of recipe and ingredient bundles (i.e. ‘meal kits’) and support the selection of additional or unexplored BCTs to influence other aspects of parental food literacy development. Using theory to inform the development of meal kit interventions seeks to magnify the opportunity to influence food literacy development. Furthermore, government at federal, state or local levels may consider subsidising the cost of MKSSs and community programmes delivering low-cost meal kits to improve equity and access for low-income families. One example of this is provided by the Minnesotan Supplemental Nutrition Assistance Program, where meal kits have recently become eligible for inclusion with Minnesotan Supplemental Nutrition Assistance Program benefits^(30)^. Evidence suggests that even with temporary exposure, meal kits may provide potential spillover effects by engaging a whole household instead of an individual^(24,25)^. Therefore, meal kit interventions may be a less resource intense way to promote food literacy compared with traditional community-based food and cooking skills interventions.

Following the BCW guide, further research could seek to explore parents’ experiences of using MKSSs in family meal provisioning using the TDF and COM-B framework to identify behavioural barriers and facilitators that could be leveraged to enhance food literacy development. Furthermore, it would be useful to explore commercial perspectives to understand how building BCTs in MKSSs to influence food literacy could support public health imperatives, while also being viable for business. Addressing the gaps in our current knowledge will aid in the development of future theoretically informed behaviour change interventions targeting food literacy and family dietary quality.

### Strengths and limitations

This study has several strengths. The application of an established framework (BCW) and validated tools (BCTTv1 and Theory and Techniques Tool) minimised potential bias related to the subjectivity of coding. Furthermore, our study adhered to best practice reporting guidelines for retrospective application of the BCTTv1 to enhance transparency and replicability^(41)^. To our knowledge, this is the first study to specify parental food literacy in behavioural terms using an established food literacy framework^(17)^. Although our study targeted parental behaviours, most food literacy behaviours specified could apply to adults without children.

There are several limitations that should be acknowledged. This study provides an exploratory analysis of the behavioural content of MKSSs and does not measure the delivery or effectiveness of BCTs. Coding of BCTs is limited to the presence or absence of a BCT in MKSS content and does not capture whether BCTs are delivered to participants (i.e. fidelity) nor BCT dose. While mapping BCTs should ideally be carried out when designing an intervention, this study applied BCTs retrospectively to an existing initiative to identify potential redesign opportunities. Parental behaviours were coded as a collective, therefore, researchers seeking to identify the influence on specific food literacy behaviours or domains would need to examine these in more detail. The pragmatic approach used to extract data from MKSSs was limited to materials accessible in a 1-week period including three recipes from each provider to ensure compatibility across MKSSs. While in some instances this included retrospective blog content from the past 12 months, this timeframe may have resulted in missed BCTs in iterative content made available to ongoing subscribers or in content available during different seasons (i.e. seasonal festivities).

Despite the potential for MKSSs to influence parental food literacy, it should be noted that our analysis did not consider the nutritional quality of the MKSS meals used nor additional meal kit services such as ‘ready-to-heat’ meals, desserts or food and beverages. The availability of these food items through the online ‘marketplace’ may reduce opportunities to positively influence food literacy-related behaviours. Therefore, the hypothetical potential of MKSSs to influence adult and child food literacy and food intakes requires further research.

### Conclusion

The novel application of behaviour change and food literacy frameworks in this study provides important insights into the opportunities that exist to harness MKSSs to promote healthy family meal provisioning and dietary behaviours. The MKSSs in this study incorporated a range of BCTs that target mechanisms of actions associated with food literacy-related behaviours. These findings provide a theory-informed understanding to support the development or optimisation of interventions incorporating ‘meal kits’ to target a range of food literacy-related behaviours. Further research is required to evaluate the potential of MKSSs to influence behaviour change or impact family food intakes.

## Supporting information

Fraser et al. supplementary material 1 Fraser et al. supplementary material

Fraser et al. supplementary material 2 Fraser et al. supplementary material

Fraser et al. supplementary material 3 Fraser et al. supplementary material

## Supplementary material

For supplementary material accompanying this paper visit https://doi.org/10.1017/S136898002300263X
