# Supplementary material for: Mapping the behaviour change potential of meal kits to positively influence parental food literacy
Source: Public Health Nutr. 2023 Dec 1;27(1):e16. doi: 10.1017/S136898002300263X (PMC10825774; doi:10.1017/S136898002300263X)
Supplement: Fraser et al. supplementary material 1 — Fraser et al. supplementary material [file S136898002300263Xsup001.docx]

Supplementary material 1

**Mapping meal kit subscription service (MKSS) components/features to the Behaviour Change Wheel (BCW) Intervention Functions and COM-B^1^ components (47).**

| **MKSS component** | **Intervention Function^2^** | **COM-B^3^** | **Types of MKSS features that are delivered via intervention functions to influence COM-B** |
| --- | --- | --- | --- |
| Subscription and meal planning | **Enablement**  Coercion  Incentivisation  Training | **Opportunity (physical)**  **Motivation (automatic & reflective)**  Capability (psychological) | - The subscription and meal planning features serve to reduce *opportunity-* and *motivation*-related barriers such as time and effort associated with meal planning and procurement. - Paid subscription may increase *motivation* to perform the behaviour through expectation of cost and reward/s. - Training to improve knowledge and cognitive skills (*capability*) required for meal planning. Reduces cognitive resources required for meal planning by offering a set weekly menu with a selected number of meals to order from. Also prepopulates recipes based on preferences for subsequent orders. - Allows users to gift a free or discounted box to friends/family which may promote positive feelings *(motivation)* for the donor. |
| Meal kit delivery  (ingredients and recipe) | **Environmental restructuring**  **Enablement**  Education  Training  Modelling | **Opportunity (physical)**  **Motivation (reflective & automatic)**  Capability (psychological) | - The provisioning of meal kits (i.e. ingredients and recipes) to users doorstep reduces the time demand (*opportunity*) for grocery shopping. - Pre-portioned/semi-prepared ingredients (e.g. coleslaw vegetables already chopped) reduce time demands (*opportunity*) for meal preparation and cooking. - The provision of resources (ingredients and recipes) restructures the home food environment to increase *opportunity* and overcome *motivation*-related barriers to cooking at home. - Increase *motivation and capability* through training and modelling of preparing home-cooked meals. - Step-by-step recipes may increase *capability* by increasing food preparation/cooking knowledge. - Increases knowledge of environmental and social consequences of using meal kits to enhance *motivation*. |
| Website content (general information, FAQs, blogs) | **Persuasion**  **Education**  Environmental restructuring | **Capability (psychological)**  **Motivation (automatic and reflective)**  Opportunity (social) | - General information and blogs written to persuade *(motivation)* or educate consumers *(capability)* to create positive beliefs and evoke positive emotions regarding meal kits. - Pictures/advertising designed to emphasise and bring attention to the performance of food preparation, cooking and consumption behaviours to make the positive consequences more memorable and increase *motivation*. - Written, verbal and visual images of environmental consequences (e.g. reduce food waste), and social consequences (e.g. locally sourced and sustainable food supply, % of ingredients sourced from Australia) provides knowledge of positive consequences of using meal kits to enhance m*otivation.* - Customer reviews on meal kit website provides information of what other people think (i.e. their approval) to increase *opportunity* and *motivation* to perform desired behaviour at home (i.e. using a meal kit to prepare home-cooked meals). - Increases *opportunity* by encouraging subscribers to restructure the social environment and get kids/partners to help plan and prepare meals. |

^1^ Capability, opportunity and motivation model of behaviour (COM-B). ^2^ **Bold** intervention functions are considered the primary functions of this MKSS component, non-bold are supporting intervention functions. ^3^ **Bold** is used to indicate the primary COM-B elements targeted by MKSS components, non-bold are additional COM-B elements that may be targeted.
