# Supplementary material for: Mapping the behaviour change potential of meal kits to positively influence parental food literacy
Source: Public Health Nutr. 2023 Dec 1;27(1):e16. doi: 10.1017/S136898002300263X (PMC10825774; doi:10.1017/S136898002300263X)
Supplement: Fraser et al. supplementary material 2 — Fraser et al. supplementary material [file S136898002300263Xsup002.docx]

Supplementary Material 2

**Presence of behaviour change techniques (BCTs) in each of the nine meal kit subscription services (MKSSs).**

| **BCTs number and label (n=35 identified)^1^** | **Meal Kit Subscription Services (MKSSs)** | | | | | | | | |
| --- | --- | --- | --- | --- | --- | --- | --- | --- | --- |
|  | **Marley Spoon^TM^** | **Dinnerly^TM^** | **HelloFresh^TM^** | **Every Plate^TM^** | **Dinner Twist^TM^** | **My Foodie Box^TM^** | **You Plate It^TM^** | **Dinner Sorted^TM^** | **Pepper Leaf^TM^** |
| 1. Goals and planning | | | | | | | | | |
| **1.1 Goal setting (behaviour)** | **x** | **x** | **x** | **x** | **x** | **x** | **x** | **x** | **x** |
| 1.2 Problem Solving |  |  | x | x | x | x | x |  |  |
| **1.4 Action planning** | **x** | **x** | **x** | **x** | **x** | **x** | **x** | **x** | **x** |
| **1.8 Behavioural contract** | **x** | **x** | **x** | **x** | **x** | **x** | **x** | **x** | **x** |
| **1.9 Commitment** | **x** | **x** | **x** | **x** | **x** | **x** | **x** | **x** | **x** |
| 2. Feedback and monitoring | | | | | | | | | |
| No BCTs identified for this category | | | | | | | | | |
| 3. Social support | | | | | | | | | |
| 3.1 Social support (unspecified) | x | x | x | x | x | x | x | x |  |
| **3.2 Social support (practical)** | **x** | **x** | **x** | **x** | **x** | **x** | **x** | **x** | **x** |
| 4. Shaping knowledge | | | | | | | | | |
| **4.1 Instruction on how perform behaviour** | **x** | **x** | **x** | **x** | **x** | **x** | **x** | **x** | **x** |
| 4.2 Information about antecedents |  |  | x | x |  |  |  |  |  |
| 5. Natural consequences | | | | | | | | | |
| 5.1 Information about health consequences | x | x | x |  | x | x | x |  | x |
| **5.2 Salience of consequences** | **x** | **x** | **x** | **x** | **x** | **x** | **x** | **x** | **x** |
| **5.3 Information about social and environmental consequences** | **x** | **x** | **x** | **x** | **x** | **x** | **x** | **x** | **x** |
| 5.6 Information about emotional consequences |  |  | x | x |  |  | x |  |  |
| 6. Comparison of behaviour | | | | | | | | | |
| 6.1 Demonstration of the behaviour | x | x | x | x | x | x | x |  |  |
| 6.2 Social comparison |  |  | x |  |  |  |  |  |  |
| **6.3 Information about others' approval** | **x** | **x** | **x** | **x** | **x** | **x** | **x** | **x** | **x** |
| 7. Associations | | | | | | | | | |
| **7.1 Prompts and cues** | **x** | **x** | **x** | **x** | **x** | **x** | **x** | **x** | **x** |
| 8. Repetition and substitution | | | | | | | | | |
| **8.1 Behavioural practice / rehearsal** | **x** | **x** | **x** | **x** | **x** | **x** | **x** | **x** | **x** |
| 8.2 Behaviour substitution |  | x | x | x | x |  | x |  | x |
| **8.3 Habit formation** | **x** | **x** | **x** | **x** | **x** | **x** | **x** | **x** | **x** |
| 9. Comparison of outcomes | | | | | | | | | |
| 9.1 Credible source | x | x | x |  | x | x | x |  | x |
| 9.2 Pros and cons |  |  | x |  |  |  | x |  | x |
| 10. Reward and threat | | | | | | | | | |
| 10.1 Material incentive (behaviour) | x | x | x | x |  | x | x | x |  |
| 10.2 Material reward behaviour | x | x | x | x |  |  |  |  |  |
| **10.3 Non-specific reward** | **x** | **x** | **x** | **x** | **x** | **x** | **x** | **x** | **x** |
| 10.4 Social reward |  |  |  |  | x |  |  |  |  |
| 10.6 Non-specific incentive |  |  |  |  |  | x |  |  | x |
| 11. Regulation | | | | | | | | | |
| 11.2 Reduce negative emotions |  |  | x | x |  | x | x | x |  |
| **11.3 Conserving mental resources** | **x** | **x** | **x** | **x** | **x** | **x** | **x** | **x** | **x** |
| 12. Antecedents | | | | | | | | | |
| **12.1 Restructuring the physical environment** | **x** | **x** | **x** | **x** | **x** | **x** | **x** | **x** | **x** |
| 12.2 Restructuring the social environment |  |  |  |  |  | x |  |  |  |
| 12.3 Avoidance/reducing exposure to cues for the behaviour |  |  |  |  | x |  |  |  |  |
| **12.5 Adding objects to the environment** | **x** | **x** | **x** | **x** | **x** | **x** | **x** | **x** | **x** |
| 13. Identity | | | | | | | | | |
| 13.2 Framing/reframing |  |  |  |  | x | x |  |  |  |
| 14. Scheduled consequences | | | | | | | | | |
| No BCTs identified for this category | | | | | | | | | |
| 15. Self-belief | | | | | | | | | |
| 15.1 Verbal persuasion about capability |  |  |  |  |  |  |  |  | x |
| 16. Covert learning | | | | | | | | | |
| No BCTs identified for this category | | | | | | | | | |
| **Total number of BCTs per MKSS** | **22** | **23** | **29** | **25** | **25** | **26** | **26** | **19** | **22** |

^1^ BCTs in **bold** indicate the BCTs that are common across all MKSSs.
