# Supplementary material for: Mapping the behaviour change potential of meal kits to positively influence parental food literacy
Source: Public Health Nutr. 2023 Dec 1;27(1):e16. doi: 10.1017/S136898002300263X (PMC10825774; doi:10.1017/S136898002300263X)
Supplement: Fraser et al. supplementary material 3 — Fraser et al. supplementary material [file S136898002300263Xsup003.docx]

Supplementary File 3

**Linking behaviour change techniques (BCTs) (n=35 unique*) identified across meal kit subscription services (MKSSs) (n=9) to Theoretical Domains Framework (TDF) and COM-B model.**

|  | | **Capability** | | | | | | **Opportunity** | | | | **Motivation** | | | | | | | | |
| --- | --- | --- | --- | --- | --- | --- | --- | --- | --- | --- | --- | --- | --- | --- | --- | --- | --- | --- | --- | --- |
|  | | Phys-ical | Psychological | | | | | Social | | Physi-cal | | Reflective | | | | | | | Automatic | |
|  | | **TDF Domains^2^** | | | | | | | | | | | | | | | | | | |
| **BCTs identified in MKSS components / features** | **Text description of the MKSS features** | Skills | | Knowledge | Memory, attention and decision processes | Behavioural regulation | Social influences | | Environmental context and resources | | Professional/social role and identity | | Beliefs about capabilities | Optimism | Beliefs about consequences | Intentions | Goals | Reinforcement | | Emotion |
| **MKSS - Subscription and meal planning features** (n=12 BCTs) | |  | | | | | | | | | | | | | | | | | | |
| **1.1 Goal setting (behaviour)** | Subscription service enters users into a written agreement (financial commitment). Meal planning and recipe selection features promote planning of weekly/fortnightly meals at least one week in advance. Email and/or text message sent in advance as a prompt/cue. Automatic pre-selection of meals. Subscription perks for referrals (i.e. send a free box to family/friends. |  | |  |  |  |  | |  | |  | |  |  |  | XX | XX |  | |  |
| **1.4 Action planning** |  |  | |  |  | X |  | |  | |  | |  |  |  |  |  |  | |  |
| **1.8 Behavioural contract** |  |  | |  |  |  |  | |  | |  | |  |  |  |  | X |  | |  |
| **1.9 Commitment** |  |  | |  |  |  |  | |  | |  | |  |  |  | X |  |  | |  |
| **7.1 Prompts and cues** |  |  | |  | XX |  |  | | XX | |  | |  |  |  | X |  | X | |  |
| **10.3 Non-specific reward** |  |  | |  |  |  |  | |  | |  | |  |  |  |  |  | XX | |  |
| **11.3 Conserving mental resources** |  |  | |  | XX | XX |  | | X | |  | |  |  |  |  |  |  | |  |
| 4.1 Instruction on how to perform behaviour* | Step-by-step instructions on how to select meals and subscribe. | XX | | XX |  |  |  | |  | |  | | XX |  |  |  |  |  | |  |
| 6.1 Demonstration of the behaviour* | Visual instructions on how to select meals, make changes to subscription. |  | |  |  |  |  | |  | |  | | XX |  |  |  |  |  | |  |
| 6.2 Social comparison | Number of meals purchased from busiest customer. |  | |  |  |  | XX | |  | |  | |  |  |  |  |  |  | |  |
| 10.4 Social reward | Email - “high five for your contribution, not just to your own tastebuds and health but for...and cutting down on food waste. You’re a superstar, [name]!” |  | |  |  |  | XX | |  | |  | |  |  |  |  |  | XX | |  |
| 10.6 Non-specific incentive | Free product/ingredient when purchasing specific meals or signing up i.e. free fruit box with every new customer order. |  | |  |  |  |  | |  | |  | |  |  |  |  |  | XX | |  |
| **Total number of BCT and TDF domain links identified in MKSS component** | | 1 | | 1 | 2 | 2 | 2 | | 2 | | 0 | | 2 | 0 | 0 | 3 | 2 | 4 | | 0 |

**Linking behaviour change techniques (BCTs) (n=35 unique*) identified across meal kit subscription services (MKSSs) (n=9) to Theoretical Domains Framework (TDF) and COM-B^1^ model (continued).**

|  | | **Capability** | | | | **Opportunity** | | | | | **Motivation** | | | | | | | |
| --- | --- | --- | --- | --- | --- | --- | --- | --- | --- | --- | --- | --- | --- | --- | --- | --- | --- | --- |
|  | | Physi-cal | Psychological | | | Social | | | Physi-cal | | Reflective | | | | | | Automatic | |
|  | | **TDF Domains** | | | | | | | | | | | | | | | | |
| **BCTs identified in MKSS components / features** | **Text description** | Skills | Knowledge | Memory, attention and decision processes | Behavioural regulation | Social influences | | Environmental context and resources | | Professional/social role and identity | | Beliefs about capabilities | Optimism | Beliefs about consequences | Intentions | Goals | Reinforcement | Emotion |
| **Delivery of meal kit** (n=5 BCTs) | |  | | | | | | | | | | | | | | | | |
| **3.2 Social support (practical)*** | Ingredients and recipes delivered to consumer’s doorstep. |  |  |  |  |  | XX | | |  | |  |  |  |  |  |  |  |
| **8.1 Behavioural practice / rehearsal** |  | XX |  |  |  |  |  | | |  | | XX |  |  |  |  | 0 |  |
| **8.3 Habit formation** |  |  |  |  | X |  |  | | |  | |  |  |  |  |  |  |  |
| **12.1 Restructuring the physical environment** |  |  |  |  |  |  | XX | | |  | |  |  |  |  |  |  |  |
| **12.5 Adding objects to the environment** |  |  |  |  |  |  | XX | | |  | |  |  |  |  |  |  |  |
| **Recipes** (n=7 BCTs) | |  | | | | | | | | | | | | | | | | |
| **4.1 Instruction on how to perform behaviour*** | Inclusion of step-by-step instructions on how to prepare the meal. | XX | XX |  |  |  |  | | |  | | XX |  |  |  |  |  |  |
| 3.1 Social support (unspecified)* | Helpline, email, social media connectivity. |  |  |  |  | XX |  | | |  | |  |  |  |  |  |  |  |
| 3.2 Social support (practical)* | Prompt to involve kids in meal preparation. |  |  |  |  | XX | XX | | |  | |  |  |  |  |  |  |  |
| 5.1 Information about health consequences* | Inclusion of health benefits of particular foods (e.g. carrots). |  | XX |  |  |  |  | | |  | |  |  | XX | XX |  |  |  |

**Linking behaviour change techniques (BCTs) (n=35 unique*) identified across meal kit subscription services (MKSSs) (n=9) to Theoretical Domains Framework (TDF) and COM-B model (continued).**

|  | | **Capability** | | | | **Opportunity** | | | | | **Motivation** | | | | | | | |
| --- | --- | --- | --- | --- | --- | --- | --- | --- | --- | --- | --- | --- | --- | --- | --- | --- | --- | --- |
|  | | Physi-cal | Psychological | | | Social | | | Physi-cal | | Reflective | | | | | | Automatic | |
|  | | **TDF Domains** | | | | | | | | | | | | | | | | |
| **BCTs identified in MKSS components / features** | **Text description** | Skills | Knowledge | Memory, attention and decision processes | Behavioural regulation | Social influences | | Environmental context and resources | | Professional/social role and identity | | Beliefs about capabilities | Optimism | Beliefs about consequences | Intentions | Goals | Reinforcement | Emotion |
| **Recipes** (continued) | |  | | | | | | | | | | | | | | | | |
| 5.3 Information about social and environmental consequences* | Percentage of ingredients in recipe sourced from Australian producers. |  | XX |  |  |  |  | | |  | |  |  | XX |  |  |  |  |
| 6.1 Demonstration of the behaviour* | Pictures/images of ingredients and cooking steps. |  | 0 |  |  |  |  | | |  | | XX |  |  |  |  |  |  |
| 8.2 Behaviour substitution* | Prompts substitution of refined carbs with fibre-rich vegetables, lean proteins and wholegrains. |  |  |  | XX |  |  | | |  | |  |  |  |  |  |  |  |
| **Total number of BCT and TDF domain links identified in MKSS component** | | 2 | 4 | 0 | 2 | 2 | 4 | | | 0 | | 3 | 0 | 2 | 1 | 0 | 1 | 0 |
| **MKSS website features** (n=18 BCTs) | |  | | | | | | | | | | | | | | | | |
| **5.2 Salience of consequences** | Images/photos of healthy, home-cooked meals, family’s cooking and eating together, fresh ingredients pre-portioned/prepped. Information about reducing food waste, supporting local farmers/businesses. Customer testimonials and free/discounted meals for friends/family. |  |  |  |  |  |  | | |  | |  |  | XX |  |  |  |  |
| **5.3 Information about social and environmental consequences*** |  |  | XX |  |  |  |  | | |  | |  |  | XX |  |  |  |  |
| **6.3 Information about others’ approval** |  |  |  |  |  | XX |  | | |  | |  | 0 |  |  |  |  |  |
| 1.2 Problem solving | Identifies barriers and enablers to cooking at home. |  |  |  | XX |  |  | | |  | | XX |  |  |  |  |  |  |
| 3.1 Social support (unspecified)* | Helpline, email, social media connectivity. |  |  |  |  | XX |  | | |  | |  |  |  |  |  |  |  |

**Linking behaviour change techniques (BCTs) (n=35 unique*) identified across meal kit subscription services (MKSSs) (n=9) to Theoretical Domains Framework (TDF) and COM-B model (continued).**

|  | | **Capability** | | | | **Opportunity** | | | | | **Motivation** | | | | | | | |
| --- | --- | --- | --- | --- | --- | --- | --- | --- | --- | --- | --- | --- | --- | --- | --- | --- | --- | --- |
|  | | Physi-cal | Psychological | | | Social | | | Physi-cal | | Reflective | | | | | | Automatic | |
|  | | **TDF Domains** | | | | | | | | | | | | | | | | |
| **BCTs identified in MKSS components / features** | **Text description** | Skills | Knowledge | Memory, attention and decision processes | Behavioural regulation | Social influences | | Environmental context and resources | | Professional/social role and identity | | Beliefs about capabilities | Optimism | Beliefs about consequences | Intentions | Goals | Reinforcement | Emotion |
| **MKSS website features** (continued) | |  | | | | | | | | | | | | | | | | |
| 4.2 Information about antecedents | Tell people about the antecedents/things that can facilitate preparing and consuming home-cooked meals, or that limit the use of takeaway/ultra-processed foods in provisioning of meals. |  | XX |  | XX |  |  | | |  | |  |  |  |  |  |  |  |
| 5.1 Information about health consequences | Inclusion of health benefits of particular foods. |  | XX |  |  |  |  | | |  | |  |  | XX | XX |  |  |  |
| 5.6 Information about emotional consequences | Text that promotes link from food/s to boosting mood, wellness, increased energy for recipient and/or family members. |  | 0 |  |  |  |  | | |  | |  |  | XX | 0 |  |  | X |
| 8.2 Behaviour substitution* | Encourages purchasing meal kits instead of takeaway or frozen convenience meals. |  |  |  | XX |  |  | | |  | |  |  |  |  |  |  |  |
| 9.1 Credible source | MKSSs promoted in media, recipes designed by professional chefs and/or dieticians. |  |  |  |  | 0 |  | | |  | |  |  |  |  |  |  |  |
| 9.2 Pros and cons | Pros and cons of using meal kits. |  |  |  |  |  |  | | |  | |  |  | XX |  |  |  |  |
| 10.1 Material incentive (behaviour) | Discounts for new customers. |  |  |  |  |  |  | | |  | |  |  | XX |  |  | XX |  |
| 10.2 Material reward (behaviour) | Discounts for returning customers. |  |  |  |  |  |  | | |  | |  |  |  |  |  | XX |  |
| 11.2 Reduce negative emotions | Messaging that promotes meal kits as reducing stress, time, energy required in meal planning and preparation. |  |  |  | XX |  |  | | |  | | X |  |  |  |  |  | XX |

**Linking behaviour change techniques (BCTs) (n=35 unique*) identified across meal kit subscription services (MKSSs) (n=9) to Theoretical Domains Framework (TDF) and COM-B model (continued).**

|  | | **Capability** | | | | **Opportunity** | | | | | **Motivation** | | | | | | | |
| --- | --- | --- | --- | --- | --- | --- | --- | --- | --- | --- | --- | --- | --- | --- | --- | --- | --- | --- |
|  | | Physi-cal | Psychological | | | Social | | | Physi-cal | | Reflective | | | | | | Automatic | |
|  | | **TDF Domains** | | | | | | | | | | | | | | | | |
| **BCTs identified in MKSS components / features** | **Text description** | Skills | Knowledge | Memory, attention and decision processes | Behavioural regulation | Social influences | | Environmental context and resources | | Professional/social role and identity | | Beliefs about capabilities | Optimism | Beliefs about consequences | Intentions | Goals | Reinforcement | Emotion |
| **MKSS website features (continued)** | |  | | | | | | | | | | | | | | | | |
| 12.2 Restructuring the social environment | Encourages all family members to get involved in meal planning, cooking and eating together. |  |  |  |  | X | XX | | |  | |  |  |  |  |  |  |  |
| 12.3 Avoidance/reducing exposure to cues for the behaviour | Advice to restructure the pantry to keep unhealthy ingredients out of sight. E.g. remove or hide salt shaker to limit the addition of salt to meals. |  |  |  |  |  | XX | | |  | |  |  |  |  |  |  |  |
| 13.2 Framing/reframing | Reframing cognitions or emotions around performing the behaviour of cooking at home by making it seem like a less onerous task. |  |  |  |  |  |  | | |  | |  |  | 0 |  |  |  | X |
| 15.1 Verbal persuasion about capability | Includes 'text' that aims to persuade participant that they can be successful at performing task. |  |  |  |  |  |  | | |  | | XX | 0 |  |  |  |  |  |
| **Total number of BCT and TDF domain links identified in MKSS component** | | 0 | 4 | 0 | 4 | 4 | 2 | | | 0 | | 3 | 2 | 7 | 2 | 0 | 2 | 3 |
|  | |  | | | | | | | | | | | | | | | | |
| **Includes 35 unique BCTs**  ***some BCTs were identified in more than one MKSS component/feature** | | 3 | 9 | 2 | 8 | 8 | 8 | | | 0 | | 8 | 2 | 9 | 6 | 2 | 7 | 3 |
| **Only including 16 BCTs common to all MKSSs (BOLD)** | | 2 | 2 | 2 | 3 | 1 | 5 | | | 0 | | 2 | 1 | 2 | 3 | 2 | 3 | 0 |

* some BCTs were identified in more than one MKSS component/feature. **Bold** BCTs (n=16) are common to all MKSSs.

Capability, opportunity and motivation model of behaviour (COM-B)

^2^Boxes with a double **XX** represent established links (stronger) between BCT and Theoretical Framework Domains (TDF) using the Theory and Techniques Tool (47), single **X** represents possible links (weaker), and boxes with a **0** represent author consensus (KF, BJJ, PL) on links present in MKSS context but inconclusive links identified in the tool.
